# Development of an automated system to measure ion channel currents using a surface-modified gold probe

**DOI:** 10.1038/s41598-021-97237-z

**Published:** 2021-09-09

**Authors:** Minako Hirano, Masahisa Tomita, Chikako Takahashi, Nobuyuki Kawashima, Toru Ide

**Affiliations:** 1 The Graduate School for the Creation of New Photonics Industries, 1955-1 Kurematsu Nishi-ku, Hamamatsu, Shizuoka 431-1202 Japan; 2 SYSTEC Corporation, 1-9-9 shinmiyakoda Kita-ku, Hamamatsu, Shizuoka 431-2103 Japan; 3 Graduate School of Interdisciplinary Science and Engineering in Health Systems, Okayama University, 3-1-1 Tsushima-naka, Kita-ku, Okayama-shi, Okayama, 700-8530 Japan

**Keywords:** Single-channel recording, Biochemical assays

## Abstract

Artificial lipid bilayer single-channel recording technique has been employed to determine the biophysical and pharmacological properties of various ion channels. However, its measurement efficiency is very low, as it requires two time-consuming processes: preparation of lipid bilayer membranes and incorporation of ion channels into the membranes. In order to address these problems, we previously developed a technique based on hydrophilically modified gold probes on which are immobilized ion channels that can be promptly incorporated into the bilayer membrane at the same time as the membrane is formed on the probes’ hydrophilic area. Here, we improved further this technique by optimizing the gold probe and developed an automated channel current measurement system. We found that use of probes with rounded tips enhanced the efficiency of channel current measurements, and introducing a hydrophobic area on the probe surface, beside the hydrophilic one, further increased measurement efficiency by boosting membrane stability. Moreover, we developed an automated measurement system using the optimized probes; it enabled us to automatically measure channel currents and analyze the effects of a blocker on channel activity. Our study will contribute to the development of high-throughput devices to identify drug candidates affecting ion channel activity.

## Introduction

Ion channel proteins play an important role in regulating various biological functions, such as generation of an action potential, neural transmission, and cell signaling^1–3^. They are found in biological membranes and control ion flow across the membranes in response to stimuli. Given the importance of the described process, ion channels have been known to be linked to serious diseases^4^. For instance, disfunction of the voltage-gated Na^+^ channels causes epilepsy or arrhythmia^5–7^; malfunction of the CFTR channel leads to cystic fibrosis, a known fatal disease^8,9^; and the activity of the P2X4 channel is associated to neuropathic pain^10,11^. Therefore, ion channel proteins are recognized as important drug targets^4,12^.

Artificial lipid bilayer recording is a technique that can be employed to measure ion channel activities electrophysiologically^13^. By measuring ion flows through ion channels in an artificial lipid bilayer membrane, the biophysical and pharmacological properties of the channels are determined with high sensitivity and accuracy. As this technique enables users to investigate channel properties in various environments by controlling the composition of solutions and lipids, the effects exerted by drug candidates on channel activity can be investigated in detail. However, utilization of this technique has been limited to research laboratories, due to its low measurement efficiency. In fact, implementation of this technique involves two time-consuming and complicated processes: preparation of the artificial lipid bilayer membranes and incorporation of the channels into the membranes^13,14^.

In order to overcome the low measurement efficiency, several approaches have been proposed. Some of them reduce the time needed to make the bilayer membrane. For example, the membranes are promptly made in a lipid solution by contacting two droplets^15–17^ or by contacting a droplet and an aqueous solution^18^. Additionally, methods exist whereby the membrane is made over a short period of time at the droplet–agarose gel interface^16^ or agarose gel–agarose gel interface^19^ employing an aqueous solution-containing agarose gel. Other techniques allow instead the incorporation rate of channels into the membrane to be enhanced, such as the technique that accelerates channel incorporation by applying a centrifugal force^20^.

**Figure 1:**
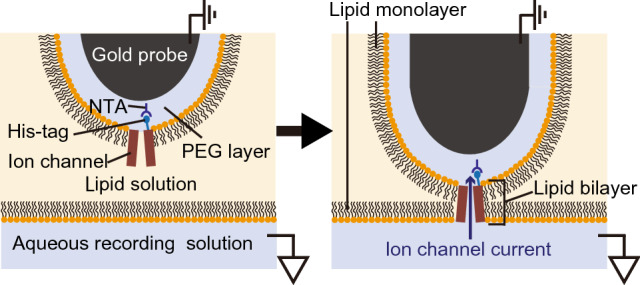
Schematic illustration of an artificial lipid bilayer single-channel recording using a gold probe. The ion channels immobilized on the surface of the probe are incorporated into a lipid bilayer membrane at the same time as the membrane is formed at the interface between the aqueous recording and lipid solution by making the probe move through a lipid solution toward an aqueous one.

Recently, we have proposed a technique that simultaneously solves both problems of making the artificial lipid bilayer membrane and incorporating channels into it^21,22^. By using gel beads or a gold probe covered with polyethylene glycol (PEG), on which ion channels are immobilized, ion channels are promptly incorporated into the bilayer membranes that are made on the beads or the probe by contacting two lipid monolayers; indeed, one such monolayer is formed on the gel bead or the probe whereas the other is formed at the interface between a lipid solution and an aqueous solution (Fig. 1). Since by this technique bilayer membrane preparation and ion channel incorporation into the membrane are achieved in a single step, channel currents are easily and efficiently measured^21–23^. Notably, as the size of the bilayer membrane increases, so does the efficiency of channel current measurements; however, as the membrane grows in size, it also becomes unstable, which is especially true in the case of membranes formed on the surface of gold probes.

Herein, we improved the efficiency of our technique by enhancing the stability of the lipid bilayer membrane formed on the gold probe. Moreover, based on the technique, we developed an automated system for the measurement of ion channel currents and the determination of the effects of a channel blocker on channel activity.

## Results and discussion

### Gold probes with a rounded tip are more efficient than those with a sharpened tip in measuring ionic currents

**Figure 2:**
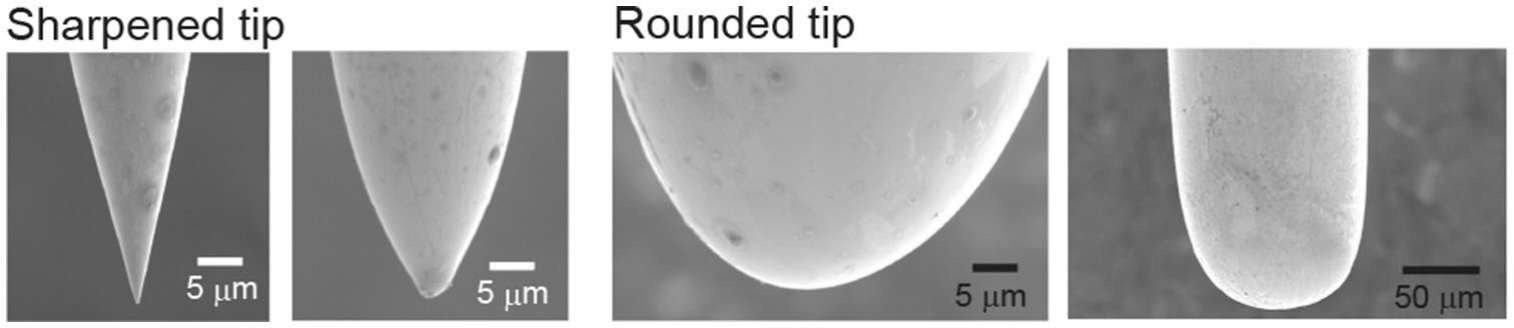
Electron micrographs of the tips of different gold probes.

We previously developed a simple technique to measure ion channel currents^22^. In this approach, by contacting a gold probe covered with a PEG-containing aqueous solution, on which channels are immobilized, with the interface between a lipid and an aqueous solution, an artificial lipid bilayer membrane comprising ion channels is formed on the surface of the tip of the probe (Fig. 1). In the present study, in order to investigate the effect of the shape of the probe tip on the efficiency of channel current measurements, we made gold probes with tips of various shapes applying gold wire electropolishing (Fig. 2). The probes were modified to anchor KcsA(E71A) channels to a PEG layer, and ionic currents were then measured.

**Figure 3:**
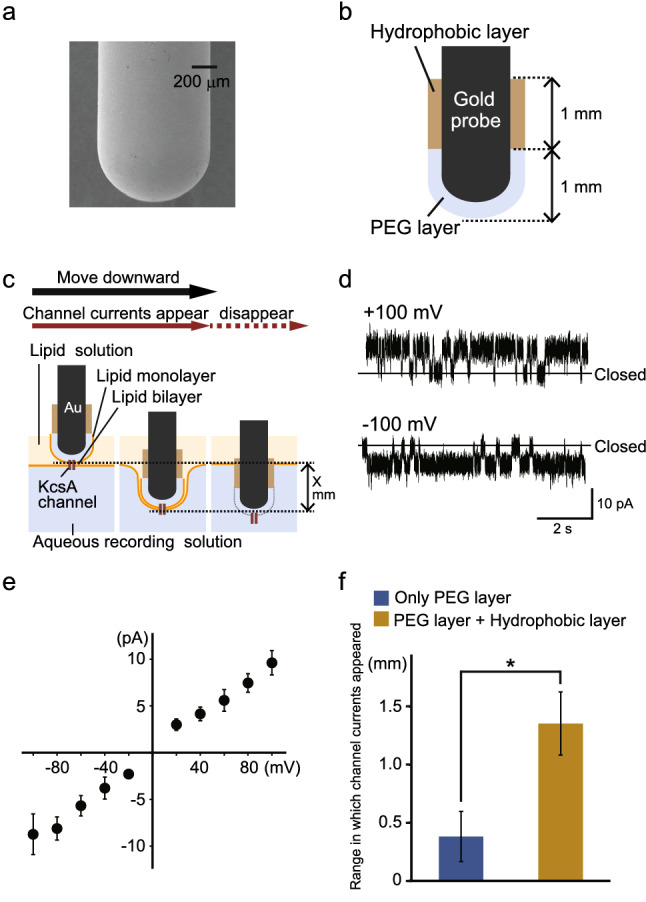
Effect of hydrophobically modifying the gold probe on the stability of the lipid bilayer membrane. **(a)** Electron micrograph of the tip of a commercially available gold probe. **(b)** Schematic depiction of a probe whose tip was modified so as to have a hydrophobic 1-octadecanethiol layer beside a hydrophilic polyethylene glycol (PEG) layer. **(c)** Procedure to investigate the stability of the lipid bilayer membrane. The bilayer membrane containing KcsA channels (KcsA(E71A)) was made at the interface between a lipid solution and an aqueous recording solution; formation of the membrane was detected by appearance of channel currents (left). The probe was then moved downward in 10-µm increments (middle), until the channel currents disappeared (right), so that the range in which the channel currents were detectable was recorded. **(d)** Representative current traces of KcsA(E71A) channels recorded in the lipid bilayer membrane consisting of POPE–POPG and *n*-decane at ± 100 mV. **(e)** Current–voltage relationship recorded for KcsA(E71A) channels (n = 7). **(f)** Length ranges wherein channel currents were detected using the POPE–POPG lipids dissolved in *n*-decane. The ranges were wider when gold probes comprising the hydrophobic layer were used. Range differences were analyzed using Welch's t-test. The bar indicates the mean standard deviation (n = 6–7). The asterisk indicates that *P* < 0.01. *POPE* 1-palmitoyl-2-oleoyl-sn-glycero-3-phosphoethanolamine; *POPG* 1-palmitoyl-2-oleoyl-sn-glycero-3-[phospho-rac-(1-glycerol)]; *POPE–POPG* lipid solution consisting of 18 mg/ml POPE and 6 mg/ml POPG.

When we used probes with rounded tips whose curvature radii had values ≥ 5 µm, we always measured KcsA(E71A) channel currents (19/19 trials with different probes). On the other hand, when we used gold probes with sharpened tips whose curvature radii had values < 5 µm, the success rate in measuring channel currents was 74% (14/19 trials with different probes). In most failed trials, large currents were measured, or the channel currents disappeared over several seconds due to the presence of a large current background noise. These phenomena were due to the penetration of the probe in the recording solution in the absence of the formation of bilayer membranes or to the rapid breakdown of unstable bilayer membranes. These results indicate that ion channel currents are measured more efficiently with rounded-tip probes (curvature radii ≥ 5 µm) than with sharpened-tip ones. We used a commercially available gold probe characterized by a rounded tip (Fig. 3a; curvature radius: 450 µm) in the following experiments.

### Modification of the surface of gold probes with a hydrophobic layer increases the stability of lipid bilayer membranes

Although we were able to measure channel currents using the gold probe, these currents often disappeared because of disruptions of the lipid bilayer membranes or the current baseline often drifted while the probe position changed a little. In order to solve this problem, we increased the stability of the lipid bilayer membrane by modifying the surface of the probe so that it had a hydrophobic area, which has high lipid affinity, besides the PEG area, on which the bilayer membrane formed. In detail, the probe was modified so that it had a hydrophilic PEG layer covering about 1 mm of the tip and a hydrophobic 1-octadecanethiol layer covering the next 1 mm of the tip (Fig. 3b).

By moving toward an aqueous recording solution and through a lipid solution (POPE–POPG dissolved in *n*-decane) the probe on which the KcsA(E71A) channels had been immobilized on the PEG layer, a lipid bilayer membrane comprising the channels was formed, and channel currents were detected (Fig. 3c). Figure 3d shows the measured channel currents. The current–voltage (I–V) relationship of the unitary current (Fig. 3e) indicated the single-channel conductance to be 103.6 ± 22.5 pS, which is *similar* to the values measured using the conventional planar bilayer method^24,25^.

After channel currents were detected, we moved the probe further in 10-µm increments until the channel currents disappeared (Fig. 3c). Figure 3f shows the ranges in which channel currents were detected as the probes moved toward the recording solution through a synthetic lipid solution (POPE:POPG = 3:1; solvent: *n*-decane); in other words, these data indicate the range in which a stable lipid bilayer membrane containing the ion channels was formed. Importantly, we were able to measure channel currents in a significantly larger length range with probes comprising the hydrophobic layer alongside the PEG layer than with probes comprising only the PEG layer. Similarly, in an asolectin solution (a mixture of phospholipids extracted from soybeans; solvent: *n*-decane), channel currents were measured in a larger length range when the hydrophobically modified probe was used than when the non-hydrophobically modified probe was used (0.417 ± 0.177 mm versus 0.078 ± 0.072 mm, respectively). These results indicate that the hydrophobic modification of the probe increased the stability of the lipid bilayer membrane containing the channels, as had also been indicated by previous studies, according to which surface modification of a material that supports the bilayer membrane affected membrane stability^26,27^.

### Use of gold probes comprising a hydrophobic layer results in the formation of thin lipid bilayer membranes

In order to further investigate the stability of the lipid bilayer membranes produced using hydrophobically modified probes, we used a lipid solution dissolved in *n*-hexadecane. Lipid bilayer membranes formed using lipids dissolved in *n*-hexadecane are known to be thinner and more fragile than those formed using lipids dissolved in *n*-decane^13^.

**Figure 4:**
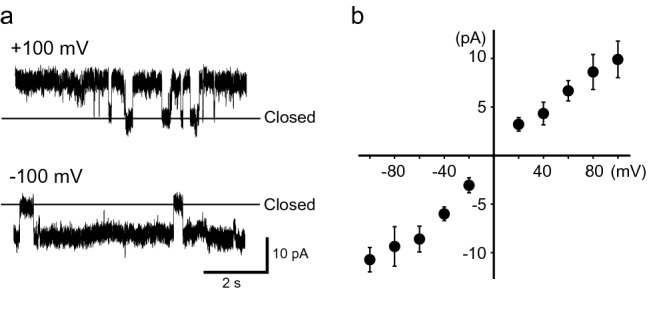
Single-channel currents measured with lipids dissolved in *n*-hexadecane using a hydrophobically modified probe. **(a)** Representative current traces of KcsA channels (KcsA(E71A)) measured in the bilayer membrane consisting of POPE–POPG dissolved in *n*-hexadecane, which is a thinner membrane than that obtained with POPE–POPG dissolved in *n*-decane, at ± 100 mV. **(b)** Current–voltage relationship of KcsA(E71A) channels (n = 7). *POPE* 1-palmitoyl-2-oleoyl-sn-glycero-3-phosphoethanolamine; *POPG* 1-palmitoyl-2-oleoyl-sn-glycero-3-[phospho-rac-(1-glycerol)]; *POPE–POPG* lipid solution consisting of 18 mg/ml POPE and 6 mg/ml POPG.

Employing a hydrophobically modified probe, we were able to measure KcsA(E71A) channel currents when we utilized the synthetic lipid solution (POPE:POPG = 3:1) dissolved in *n*-hexadecane (Fig. 4); in this case, the single-channel conductance calculated from the I–V relationship was 127.6 ± 11.4 pS, which was slightly larger than that measured in *n-*decane (Fig. 3). Because it has been previously shown that KcsA channel conductances are affected by the compositions of phospholipids^28^, solvents dissolving the lipids might also influence the activities. On the other hand, no channel currents were detected when a non-hydrophobically modified probe was used in an *n*-hexadecane lipid solution (n = 12). These results support the evidence that the lipid bilayer membrane formed on the PEG layer was stabilized by the presence of the hydrophobically modified area besides the PEG layer.

This technique has the potential to allow the activity of various human channels to be measured efficiently. Notably, the single-channel current of some biological channels, for example the nicotinic acetylcholine receptor channel, is known to be detectable in artificial lipid bilayer membranes containing *n*-hexadecane or in membranes formed in the absence of a hydrocarbon solvent^29–32^; however, few studies have been published wherein currents were measured in membranes containing *n*-decane. In fact, we have tried to measure the current across the nicotinic acetylcholine receptor channel using membranes containing *n*-decane, but we have been unsuccessful. Since membranes containing *n*-decane are thicker than those containing *n*-hexadecane, and they are more than twice as thick as biological membranes, it has been speculated that the thickness of the membrane affects the structural changes in the ion channels. As our technique using the hydrophobically modified probes enabled us to measure channel currents in bilayer membranes containing *n*-hexadecane more simply and efficiently than the conventional method, the technique should afford the efficient measurement of the activity of human channels.

### Automated system for the measurement of ion channel activities

**Figure 5:**
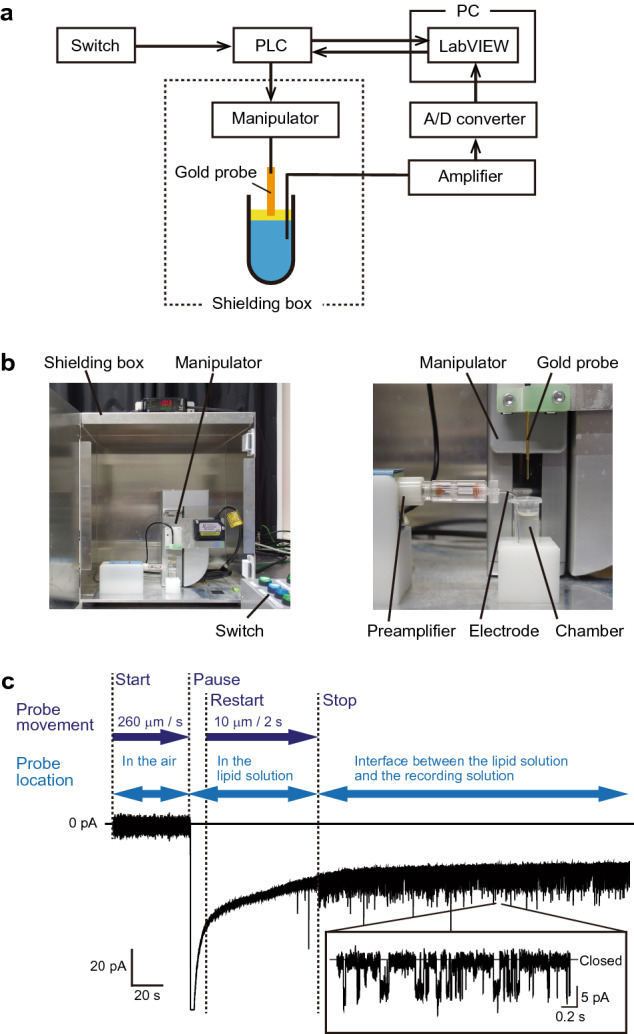
The automated system for the measurement of ion channel currents. **(a)** Flow diagram of the automated measurement system using the gold probe. **(b)** Photograph of the measurement system and the shielding box that contains it (left) and blow-up of the system (right). **(c)** Typical current trace automatically measured using the system at − 60 mV. The system moved the probe until channel currents were automatically detected.

We developed an automated measurement system using the gold probes to monitor ion channel activity. The system consists of a driving device and LabVIEW software controlled by a PLC (Fig. 5a,b). The driving device moves the gold probe, and the LabVIEW software detects any channel currents. Notably, the movement of the probe determined by the driving device is feedback-regulated by the currents detected by LabVIEW through the PLC.

Figure 5c shows a typical current trace recorded by a system using a gold probe having a hydrophobic layer beside the PEG layer. Once the start button was pressed, the driving device began moving the probe at a rate of 260 µm/s. When the PLC detected a large current flow triggered by the probe contacting the lipid solution, the system stopped probe movement for 10 s. Afterwards, the driving device moved the probe by 10-µm increments at 2-s intervals, and the LabVIEW software analyzed currents until it detected channel currents. In detail, the software was configured to recognize the current which changed by more than 2 pA within 10 ms and then returned to its original value within 0.01–1 s as a channel current. KcsA(E71A) channel currents were automatically detected. In the cases in which the channel currents were ≥ 5 pA, the system detected the currents perfectly (*n* = 30). Conversely, although we set up the system to recognize the channel currents of ≥ 2 pA, the currents < 5 pA were hard to detect; channel currents that are ≥ 4 pA and < 5 pA were detected at 67% (*n* = 6), and the system did not detect currents that were < 4 pA at all (*n* = 17). The failures to detect currents were likely caused by the difficulty of distinguishing channel currents from background noise at these low current values. In addition, the system misidentified background current spikes as channel currents, which caused the probe movement to be interrupted. In order to increase the success rate of these measurements, the background current noise needs to be lowered and the sensitivity of the system needs to be increased.

The membranes containing the channels made by the automated system were stable; 95% of the channel currents were measured for longer than 3 min (*n* = 29), and breakage of the lipid bilayer membranes was not observed in any experiment. We investigated the number of channels that were incorporated into the lipid bilayer membrane and single-, double-, or multiple-channel currents were detected at 71%, 20%, and 9%, respectively (*n* = 41), and there was no recording that detected more than five channels. Therefore, the channel properties were determined at a single-channel level.

The developed automated system enabled us to detect channel currents in several minutes. In the conventional artificial lipid bilayer recording technique, it takes several tens of minutes or several hours to measure channel currents because it takes a long time to both make the lipid bilayer membrane (several minutes or more) and incorporate the ion channels into the membrane (from approximately ten minutes to several hours) although it depends on the type of channel. In addition, for a commercially available system to measure channel current in the artificial membrane using giant unilamellar vesicles, it has been reported that average recording times are 1–3 times per day^33^. Our system provides much higher measurement efficiency than the other techniques.

### Automated measurement system detects the effects of an ion channel blocker

Using the developed automated measurement system, we analyzed the effects exerted by tetraethylammonium (TEA), a known channel blocker, on the activity of KcsA(WT) channels^34^. In this study, the recording solution is on the extracellular side of the KcsA channel, as the N-terminus located at the intracellular side of KcsA(WT) channels were anchored at the probe; the effects of TEA from the channels’ extracellular side on the channels were thus measured. The automated system was operated at a high enough voltage to produce detectably large channel currents. Once the channel currents had been detected, we measured the current at various voltages.

**Figure 6:**
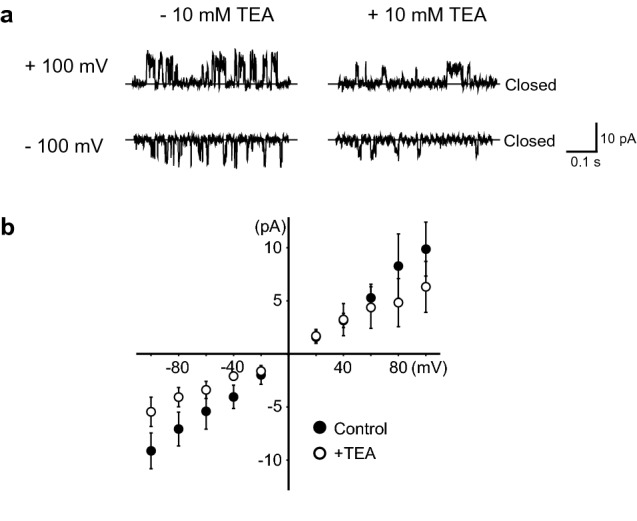
Analysis of effects of tetraethylammonium (TEA) on the activity of wild-type KcsA channels (KcsA(WT)) using the automated measurement system. **(a)** Representative current traces of KcsA(WT) channel measured in the bilayer membrane consisting of POPE–POPG and *n*-decane in the indicated conditions. The single-channel currents were smaller in the presence of TEA than in its absence. **(b)** Current–voltage relationship for the KcsA(WT) KcsA channel (n = 7) in the absence (Control) and presence (+ TEA) of 10 mM TEA (n = 5). *POPE* 1-palmitoyl-2-oleoyl-sn-glycero-3-phosphoethanolamine; *POPG* 1-palmitoyl-2-oleoyl-sn-glycero-3-[phospho-rac-(1-glycerol)]; *POPE–POPG* lipid solution consisting of 18 mg/ml POPE and 6 mg/ml POPG.

We measured channel activity in the absence and presence of 10 mM TEA using the automated system, and we detected a reduction in single-channel currents in the presence of TEA (Fig. 6). In the absence of TEA, the conductance calculated from the I–V curve had a value of 90.2 ± 26.4 pS; by contrast, the value was 52.2 ± 9.1 pS in the presence of TEA. These results indicate that the developed automated system enabled us to analyze the effect of TEA on the activity of the KcsA channel; in other words, the system has the potential to identify drug candidates that influence the activity of specific ion channels associated with serious diseases. In the future, by improving this system to obtain a multi-channel system, it should become possible to detect drug candidates that affect the activity of specific ion channels with high efficiency.

## Methods

### Purification of KcsA channels

Wild-type KcsA channel (KcsA(WT)) and its E71A mutant (KcsA(E71A)), both of which comprise a His-tag at their N-terminus, were purified as previously described^24,35^. We used KcsA(WT) in the experiments whereby the effect of TEA on KcsA(WT) activity was investigated; on the other hand, we used KcsA(E71A) in the other investigations whose single-channel current was clear due to high open probability.

### Electrochemical etching of gold wires

We sharpened the tips of gold wires by electrochemical etching as previously described^22,36^. Although every 0.25 mm-diameter gold wire was etched by the same procedure, gold wires with different tip shapes were obtained (Fig. 2). We observed the etched gold wires and measured their curvature radii by scanning electron microscopy (VE-8800, Keyence, Japan).

### Chemical modification of gold probes

We chemically modified the tips of gold probes sharpened by electrochemical etching or of commercially available gold probes (contact probe, KS-100 305 090 A 2000, INGUN, Germany) following a previously reported method with some modifications^22^. Briefly, these probes were cleaned by ozone ashing (UV Ozone Cleaner, UV253, Filgen, Japan) for 1 h; their tips (over 2 mm in length) were then immersed in 2 mM thiol-PEG solution (Thiol PEG (SUNBRIGHT ME-050SH, Mw 5000, YUKA SANGYO, Japan):Poly(ethylene glycol) 2-mercaptoethyl ether acetic acid (757837, Mn 5000, Sigma-Aldrich, USA) = 1000:1) for 2 h, so as to obtain probes whose tips were covered by a PEG layer. When making gold probes that had a hydrophobic layer, in addition to the PEG layer, the tips of the probes were first immersed to a depth of about 1 mm in 2 mM thiol-PEG solution for 2 h and subsequently to a depth of about 2 mm in 5 mM 1-octadecanethiol for 2 h, using a micromanipulator. Following PEG or PEG plus 1-octadecanethiol modification, the tips of the probes were incubated in a solution of 200 mM 1-ethyl-3-(3-dimethylaminopropyl)carbodiimide HCl (EDC, Thermo Fisher Scientific, USA) and 50 mM *N*-hydroxysulfosuccinimide (sulfo-NHS, Tokyo Chemical Industry, Japan) for 1 h, and then they were modified with 50 mM *N*-(5-amino-1-carboxypentyl)iminodiacetic acid (AB-NTA, Dojindo, Japan) for 1 h. After this step of the procedure, Ni^2+^ was bound to the NTA group by immersing the tips of the probes in 10 mM NiCl_2_ for 0.5 h. Finally, the tips of the probes were incubated in a solution of 0.5 µg/ml KcsA channels having the His-tag in KcsA buffer (100 mM KCl, 5 mM *n*-decyl-β-d-maltoside, 20 mM Tris–HCl (pH 7.0)) for 5 min, so as to immobilize the channels on the probes’ surfaces as a result of the interaction between the Ni^2+^–NTA moieties and the His-tag. The probes were then washed with Wash buffer (KcsA buffer plus 20 mM imidazole) to remove free KcsA channels and subsequently immersed in a recording buffer for several minutes, before carrying out the measurements.

### Formation of lipid bilayer membranes and incorporation of KcsA channels

The lipid bilayer membranes containing KcsA channels were obtained as previously reported^22,36^. Briefly, we poured 1.2 ml of an aqueous recording solution (200 mM KNO_3_ or KCl, 10 mM MES (pH 4.0)), into a 1.5-ml microtube; subsequently, 150 µl of a lipid solution consisting either of 18 mg/ml 1-palmitoyl-2-oleoyl-sn-glycero-3-phosphoethanolamine (POPE, Avanti, Polar Lipids, USA) and 6 mg/ml 1-palmitoyl-2-oleoyl-sn-glycero-3-[phospho-rac-(1-glycerol)] (POPG, Avanti, Polar Lipids, USA) in *n*-decane or n-hexadecane, or 24 mg/ml asolectin extracted from soybeans (Sigma-Aldrich, USA) in *n*-decane were layered over the recording solution. The gold probe modified with the PEG layer on which KcsA channels had been attached was made to move into the recording solution through the lipid solution using a manipulator (uM-3A-S1, SENSAPEX, Finland) or an electric actuator (RCP6-SA4R-WA-35P-2, IAI, Japan). As a result, the lipid bilayer membrane was formed at the interface between the two solutions. At this stage, KcsA channels were spontaneously incorporated into the membrane. The membrane capacitance measured using the commercially available gold needle (curvature radius: 450 µm) was approximately 1 nF, which indicated that the bilayer membrane area was estimated to be approximately 0.25 mm^2^, assuming that the electrical capacitance of the bilayer was 0.4 µF/cm^2^^13^.

### Ionic current recording

Ionic currents were measured using a patch-clamp amplifier (CEZ2400, NihonKohden, Japan). The currents were digitized with Digidata 1440 (Molecular Devices, USA) and sampled at 10 kHz using the Clampex 10.6 Software (Molecular Device). The gold probe was held at virtual ground, and the recording solution was connected to the amplifier. Polarization effects of the electrodes were corrected by removing voltage offsets with the amplifier just before measurements. Channel currents were analyzed using the Clampfit 10.6 software (Molecular Device). Current traces and I–V curves display outward currents as positive and inward currents as negative.

### Automated measurement system for ion channel activities

The automated system for the measurement of ion channel activity consisted of an electric actuator (RCP6-SA4R-WA-35P-2, IAI, Japan) and LabVIEW software (National Instruments, USA). Both components were controlled by a programmable logic controller (PLC, FX5U-32MT/DS, FX5-4AD-ADP, Mitsubishi Electric, Japan). The gold probe comprising ion channels was attached to the actuator, and its position was monitored by a laser displacement sensor (IL-S 100, KEYENCE, Japan). Ionic currents detected by the patch-clamp amplifier (CEZ2400, NihonKohden, Japan) were monitored using the PLC or LabVIEW software. The LabVIEW software analyzed the ionic current data after sampling at 1 kHz.

The automated channel current measurements were performed as follows: first, the gold probe comprising ion channels was attached to the actuator, and a chamber (a 1.5-ml microtube) containing a recording solution (200 mM KNO_3_, 10 mM MES (pH 4.0)) was placed under the probe. In the experiment conducted to investigate the effect of TEA on KcsA(WT) channel activity, a recording solution to which had been added TEA to a final concentration of 10 mM was utilized. The recording solution was then covered with a lipid solution of 18 mg/ml POPE and 6 mg/ml POPG (POPE–POPG) dissolved in *n*-decane or 24 mg/ml asolectin dissolved in *n*-decane. The automated channel current measurements were started by pressing a start button; as a result, the actuator began to descend at a rate of 260 µm/s. Subsequently, when a large transient current induced by contact between the probe and the lipid solution was detected by the PLC, the PLC sent a signal to the actuator to temporally stop motion for 10 s. Afterwards, the actuator descended in 10-µm steps, and the LabVIEW software began analyzing the current at every step. The software determined whether channel currents were detected within 2 s, and the results were returned to the PLC. When the current changed by more than 2 pA within 10 ms and then returned to its original value within 0.01–1 s, the LabVIEW software recognized the current as a channel current, and the PLC terminated the actuator’s movement. The detected channel currents were then recorded using Clampex 10.6 Software. In order to reduce the background current noise, the gold probes were immersed in the lipid solution for over 1 h before the experiment, a procedure that afforded the tight arrangement of lipid molecules on the probe’s surface.
